# Quantitative assessment of radiotherapy-induced myocardial damage using MRI: a systematic review

**DOI:** 10.1186/s40959-023-00175-0

**Published:** 2023-05-18

**Authors:** Alireza Omidi, Elisabeth Weiss, Cory R. Trankle, Mihaela Rosu-Bubulac, John S. Wilson

**Affiliations:** 1 Department of Radiation Oncology, Virginia Commonwealth University Health System, Richmond, VA 23219 USA; 2 Department of Biomedical Engineering, Virginia Commonwealth University, Richmond, VA USA; 3 Department of Internal Medicine, Virginia Commonwealth University Health System, Richmond, VA USA

**Keywords:** Radiotherapy, Myocardial toxicity, Magnetic resonance imaging

## Abstract

**Purpose:**

To determine the role of magnetic resonance imaging (MRI)-based metrics to quantify myocardial toxicity following radiotherapy (RT) in human subjects through review of current literature.

**Methods:**

Twenty-one MRI studies published between 2011-2022 were identified from available databases. Patients received chest irradiation with/without other treatments for various malignancies including breast, lung, esophageal cancer, Hodgkin’s, and non-Hodgkin’s lymphoma. In 11 longitudinal studies, the sample size, mean heart dose, and follow-up times ranged from 10-81 patients, 2.0-13.9 Gy, and 0-24 months after RT (in addition to a pre-RT assessment), respectively. In 10 cross-sectional studies, the sample size, mean heart dose, and follow-up times ranged from 5-80 patients, 2.1-22.9 Gy, and 2-24 years from RT completion, respectively. Global metrics of left ventricle ejection fraction (LVEF) and mass/dimensions of cardiac chambers were recorded, along with global/regional values of T1/T2 signal, extracellular volume (ECV), late gadolinium enhancement (LGE), and circumferential/radial/longitudinal strain.

**Results:**

LVEF tended to decline at >20 years follow-up and in patients treated with older RT techniques. Changes in global strain were observed after shorter follow-up (13±2 months) for concurrent chemoradiotherapy. In concurrent treatments with longer follow-up (8.3 years), increases in left ventricle (LV) mass index were correlated with LV mean dose. In pediatric patients, increases in LV diastolic volume were correlated with heart/LV dose at 2 years post-RT.

Regional changes were observed earlier post-RT. Dose-dependent responses were reported for several parameters, including: increased T1 signal in high-dose regions, a 0.136% increase of ECV per Gy, progressive increase of LGE with increasing dose at regions receiving >30 Gy, and correlation between increases in LV scarring volume and LV mean/V10/V25 Gy dose.

**Conclusion:**

Global metrics only detected changes over longer follow-up, in older RT techniques, in concurrent treatments, and in pediatric patients. In contrast, regional measurements detected myocardial damage at shorter follow-up and in RT treatments without concurrent treatment and had greater potential for dose-dependent response. The early detection of regional changes suggests the importance of regional quantification of RT-induced myocardial toxicity at early stages, before damage becomes irreversible. Further works with homogeneous cohorts are required to examine this matter.

## Introduction

Cancer and cardiovascular disease are the two most common causes of death globally, taking an estimated 9.6 million and 17.9 million lives each year, respectively [1, 2]. The convergence of these two prevalent pathologies is the focus of the growing field of cardio-oncology. While overall cancer survival rates have been increasing, identifying and managing potential side effects of cancer therapy remain a significant challenge, including those due to chemotherapy (medications designed to eliminate cancer cells), targeted cancer therapy (medications to inhibit specific molecules associated with carcinogenesis) [3], immunotherapy (novel therapeutic agents to retrain the affected immune system and restore their anti-cancer function) [4], and radiotherapy (use of ionizing radiation to eliminate cancer cells). These side effects include cardiac and vascular complications that are collectively referred to as cardiovascular toxicity (e.g., heart failure, myocardial ischemia/infarction, arrhythmias) [3–6], which may occur acutely [7] or chronically after initiating therapy [8, 9].

In this review, we focus our primary attention on RT-induced myocardial toxicity (MCT) evaluated by cardiac magnetic resonance (CMR) imaging. To provide background, a brief description of the basic pathophysiology and risk factors for developing RT-induced myocardial injury is presented first, followed by a brief review of currently available imaging modalities for evaluating cardiovascular toxicity. We then discuss in detail key CMR-based quantitative metrics to assess MCT and conclude with a discussion on the benefits of each metric, the need for high quality image registration, and suggestions for future work.

### Pathophysiology

RT-induced MCT results from diverse mechanisms leading to multiscale effects on cardiac structure and function. In brief, on the subcellular level, oxidative stress and the inflammatory response within endothelial cells are responsible for the production of numerous cytokines (e.g. interleukin [IL]-1, IL-6, IL-8, IL-18, tumor necrosis factor [TNF]-α)) associated with RT-induced cardiotoxicity (e.g., fibrosis) [10, 11]. High concentrations of free radicals (primarily reactive oxygen species (ROS)) produced by RT dysregulate enzymatic activity, increase lipid peroxidation, and induce cellular damage and/or death. Indeed, ROS can increase hypertrophy and fibrosis and trigger the release of calcium leading to cellular apoptosis and necrosis [11]. ROS also impair mitochondrial function. Notably, 40% of the volume of cardiomyocytes consist of mitochondria; hence, mitochondrial dysfunction can significantly affect cardiac function [12]. Several common pathways in the development of cardiovascular toxicity are discussed elsewhere [10].

On the cellular and matrix level, histological evaluation of MCT is frequently associated with fibrosis within the myocardium [13]. Though cardiac myocytes are relatively resistant to direct low-to-moderate doses of radiation, myocardial damage and proinflammatory changes may still be induced indirectly through microvascular and macrovascular damage [14, 15]. Inflammatory cells and cytokines promote the differentiation of vascular smooth muscle cells into myofibroblasts, resulting in increased production of collagen. In addition, RT-induced endothelial injury can lead to narrowing of the capillaries and a drop in the effective ratio of blood vessels to myocytes, thus increasing the risk of myocyte injury and death. Eventually, the interstitium and damaged myocytes are replaced by collagenous fibrotic tissue, leading to focal or global myocardial stiffening [16]. For example, studies of MCT in irradiated rabbits demonstrated a neutrophilic infiltrate in all layers of the heart within six hours post-radiation. After two days, slight progressive fibrosis was noted in the pericardium and myocardium. Luminal obstruction was also accompanied by thrombogenesis, further promoting ischemia, myocardial cell death, and fibrosis [14, 17].

The cumulative effects of these subcellular, cellular, and matrix changes are responsible for early (e.g., acute restrictive pericarditis [7]) and late cardiac damage (e.g., diastolic dysfunction [8]) associated with RT, with a median development time of 10-15 years for long-term toxicities [14].

### Risk factors for developing MCT

In addition to the length of time since RT, several other risk factors have been shown to increase the risk of developing RT-induced MCT [18]. First, pre-existing cardiovascular disease like coronary artery disease, ischemic heart disease, hypercholesterolemia and certain risk factors like smoking and diabetes [15] have been shown to increase MCT risk [19]. Second, evidence suggests that certain demographics may be at higher risk, including a young age (<20-year-old) at the time of RT [20], females [21, 22], and black race [23]. Third, the risk of MCT is also linked to radiation dose and technique. Higher mean heart dose (>15 Gy) [24] and older techniques of radiation delivery [25] have been shown to increase the chance of myocardial damage following RT. Finally, the presence of concurrent treatments (e.g., chemotherapy and/or immunotherapy) may also affect the risk of cardiac damage both as an independent additive effect and potentially compounding the effect of RT-induced damage [20]. These combined therapeutic regimens can make it difficult to distinguish individual effects of highly localized RT in the presence of underlying global dysfunction due to systemic therapies without careful evaluation of spatial correlations of quantitative metrics of cardiovascular function and focal radiation dose.

### Detecting and serially monitoring cardiovascular toxicity

Due to the clinical significance of developing cardiovascular complications and the extended period of time over which they may occur, careful and repeated monitoring for evidence of MCT is essential. The variably progressive nature of RT-induced MCT, which can lead to irrevocable pathological remodeling if unmitigated, creates a specific clinical need for detecting cardiovascular dysfunction at its earliest (and potentially reversible) stages when medical interventions might provide the greatest benefit. Early complications like constrictive pericarditis (preceded by shortness of breath symptom) may appear within one year after RT [7] with potentially reversible results if treated early while late effects like coronary artery disease and risk of sudden cardiac death may not manifest until 10-15 years later (with myocardial infarction and angina symptoms) [18, 26] with a much lower possibility of successful medical interventions. Notably, early signs may be focal in nature, and thus may require detection methods capable of distinguishing the function of key substructures of the cardiovascular system. As a result, one of the primary means for these patient-specific assessments is through clinical imaging. Numerous studies over the past years have monitored the effects of various cancer treatments on the structural and functional features of the cardiovascular system, either cross-sectionally or longitudinally, using multiple imaging approaches. Below is a summary of the most commonly utilized modalities.

#### Echocardiography

Both highly available and generally affordable, echocardiography is commonly used to measure cardiac left ventricular ejection fraction (LVEF), strain, and strain rate, as well as diastolic function, valvular function, vascular flow, and large vessel pathology [27]. Despite its excellent safety profile, echocardiography suffers from some notable limitations, including: poor acoustic windows [28] (i.e., finding unobstructed views of all relevant structures free from interference from bone or lung tissue), insensitivity to small LVEF changes, underestimation of LVEF, intra/inter-reader variability [29, 30], and challenges in longitudinal studies due to temporal variability [27, 31]. Using 3D echocardiography and contrast improves some of the limitations [31]; however, challenges persist in providing assessments of underlying constitutive remodeling of the myocardium (e.g., fibrosis, edema).

#### Nuclear Imaging

A second imaging technique for assessing MCT by measuring LVEF is through multiple-gated acquisition (MUGA) nuclear scanning [28]. MUGA is more reproducible than 2D echocardiography [32, 33]; however, MUGA cannot assess tissue characteristics, the wall thickness or valvular morphologies associated with LV dysfunction [34].

Cardiac positron emission tomography (PET) is another form of nuclear imaging which has been proven to be an effective modality for assessment of myocardial perfusion, fibrosis, and inflammation with higher sensitivity to early changes compared to echocardiography [35]. However, different tracers have shown strengths and weaknesses in detection of different myocardial abnormalities. For example, fluorodeoxyglucose and 13N-ammonia are utilized to localize areas of inflammation and perfusion, respectively [36]. Therefore, the serial monitoring of myocardial dysfunction using a single tracer may prove inadequate given the wide range of myocardial abnormalities which may occur after RT.

In general, nuclear imaging has the drawback of requiring radiation exposure to the patient, though the added risk to cancer patients (who on average already receive higher doses of radiation as part of their treatment) is less clear. Thus, nuclear imaging can be a beneficial tool in the diagnosis of MCT after a thoughtful risk-benefit analysis for a given patient.

#### Cardiac computed tomography (CT)

Cardiac computed tomography (CT) is a third modality used for structural and functional cardiac assessments and has particular strength in evaluating coronary artery disease [27]. Other particular advantages of cardiac CT include high spatial resolution, short exam time, and high sensitivity for calcified tissues, making it a reliable non-invasive imaging modality for assessment of coronary arteries and functional analyses of the heart [37]. CT does require ionizing radiation, which must be considered when using this modality for serial follow-up and/or young patients. However, it has fewer contraindications due to implanted hardware (unlike magnetic resonance imaging (MRI)) and is more frequently used in urgent/emergent settings than MRI.

#### Cardiac magnetic resonance (CMR)

Finally, a fourth imaging modality, and the focus of this review, is cardiac magnetic resonance. CMR has been described as the gold standard method to assess LV function [34]. CMR can precisely measure dimensions of cardiac chambers and myocardial mass with minimal geometric assumptions, providing highly accurate and reproducible measures of LV stroke volume and LVEF. Additionally, global longitudinal strain (GLS) and global circumferential strain (GCS) can be measured through feature tracking and other methods. However, newer quantitative CMR techniques also have the potential to detect regional and subclinical myocardial changes before the onset of global dysfunction. Overall, its capacity for tissue characterization, excellent reproducibility/accuracy compared to echocardiography, lack of ionizing radiation, and ability to conduct both global and regional structural/functional assessments make CMR a well-suited technique to monitor the cardiovascular function of patients during and after cancer therapy in order to guide, and if necessary modify, the treatment plan [28, 31, 34, 38].

Some of the cardiovascular metrics capable of evaluation by CMR include: the dimensions/volume/mass of the heart and relevant substructures (e.g., LV, right ventricle (RV), left atrium (LA), and right atrium (RA)), tissue characterization (e.g., fibrosis, edema, scar) using T1/T2 parameters and LGE, regional and global quantification of myocardial contraction using various 2D/3D strain techniques (e.g., tissue tracking) over circumferential, radial and longitudinal directions, and quantification of flow in terms of velocity, pressure drop, and shear stress on the LV endocardium and aortic wall [31, 34, 39]. Despite its many advantages, it should be noted that CMR is generally more expensive, is less widely available, takes longer to complete, and is associated with a greater number of contraindications (claustrophobia, MR-incompatible implants, allergy to contrast agents, pregnancy, etc.) than some of the other modalities. Furthermore, it can be more challenging for sick and older patients who may struggle with performing the breath-hold associated with some of the CMR sequences [38]. In addition, when specifically applying CMR for evaluation of dose-dependent RT-induced cardiotoxicity, the challenge of spatially correlating the traditionally CT-based spatially heterogeneous dosimetry map with metrics derived from various 2D and 3D CMR images must be addressed through careful image registration.

## MRI-based metrics evaluation

The focus of this review will be on studies that have used CMR techniques to quantify MCT metrics in patients who have undergone RT. Though clearly important, a full review of chemotherapy-induced cardiotoxicity (without RT) and evaluation of cardiotoxicity metrics using other imaging modalities or biomarkers is beyond the scope of this article. We point the interested reader to numerous informative prior reviews [12, 40–42]. Searching PubMed, Google Scholar, ScienceDirect, and Google, with the following phrases: ‘radiotherapy and cardiotoxicity using MRI’ and ‘Using MRI to detect cardiotoxicity after radiotherapy’ resulted in ‘54’ and ‘11’, ‘9980’ and ‘12000’, ‘1128’ and ‘882’, ‘154000’ and ‘95300’ papers, respectively. Papers were sorted by relevance using automatic filtering and the first 1000 papers from each database were selected for initial screening. Next, papers were excluded based on the title and/or the abstract review leading to 1560 eligible papers for comprehensive review. Final exclusion was made based on lack of RT in the treatment plan, missing MRI-based metrics for cardiac function assessments, dismissing myocardial evaluation in MRI-based studies, and the use of non-human subjects for RT-induced MCT measurements. In total, 21 papers ranging from 2011-2022 were identified that specifically focused on RT-induced MCT evaluation in human studies using CMR.

**Fig. 1:**
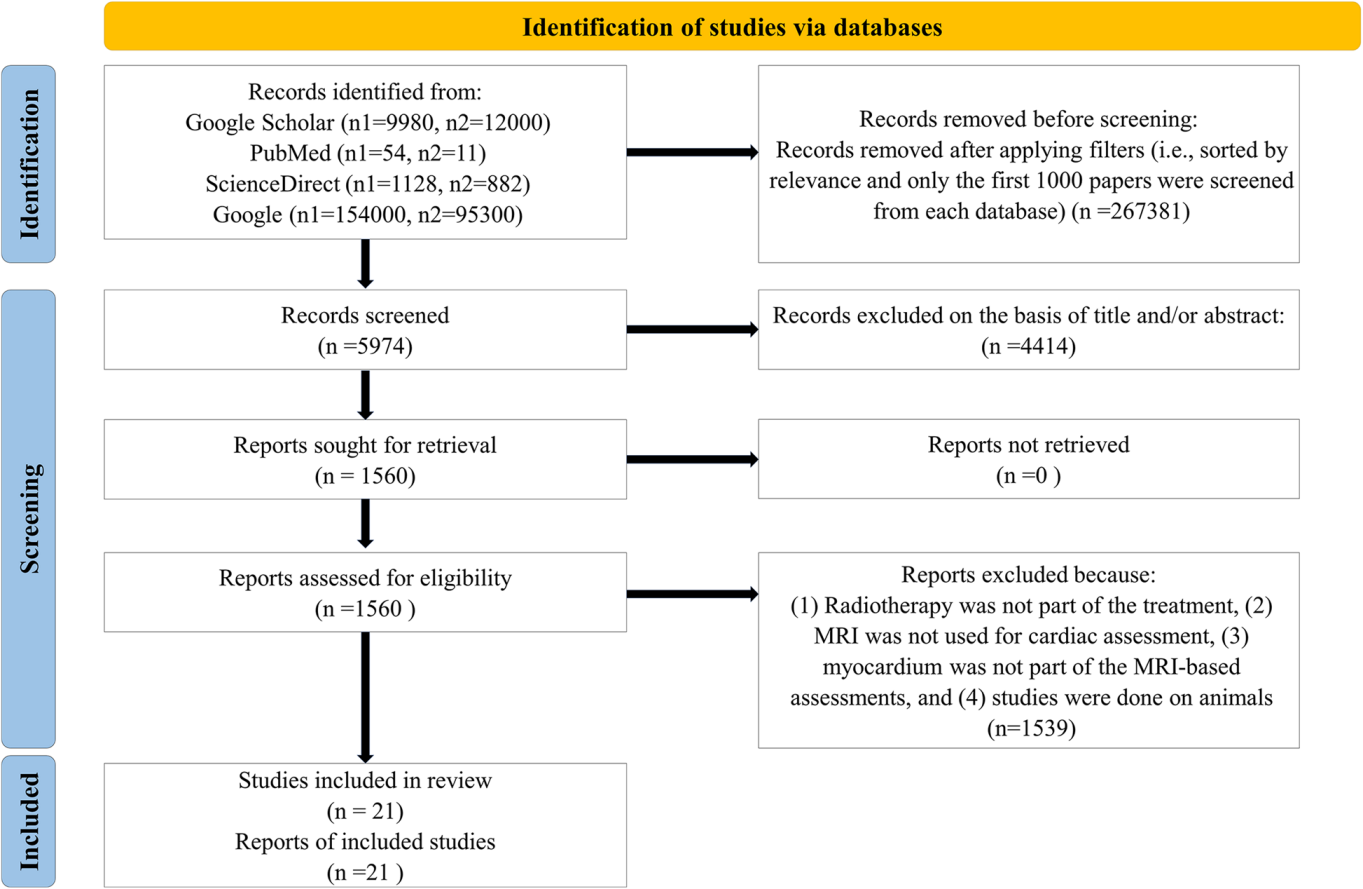
Flow chart for identification of studies via databases following a PRISMA 2020 flow diagram [43] modified based on inclusion/exclusion criteria in this review paper. The notations n1 and n2 refer to phrase searches ‘radiotherapy and cardiotoxicity using MRI’ and ‘using MRI to detect cardiotoxicity after radiotherapy’, respectively

**Fig. 2:**
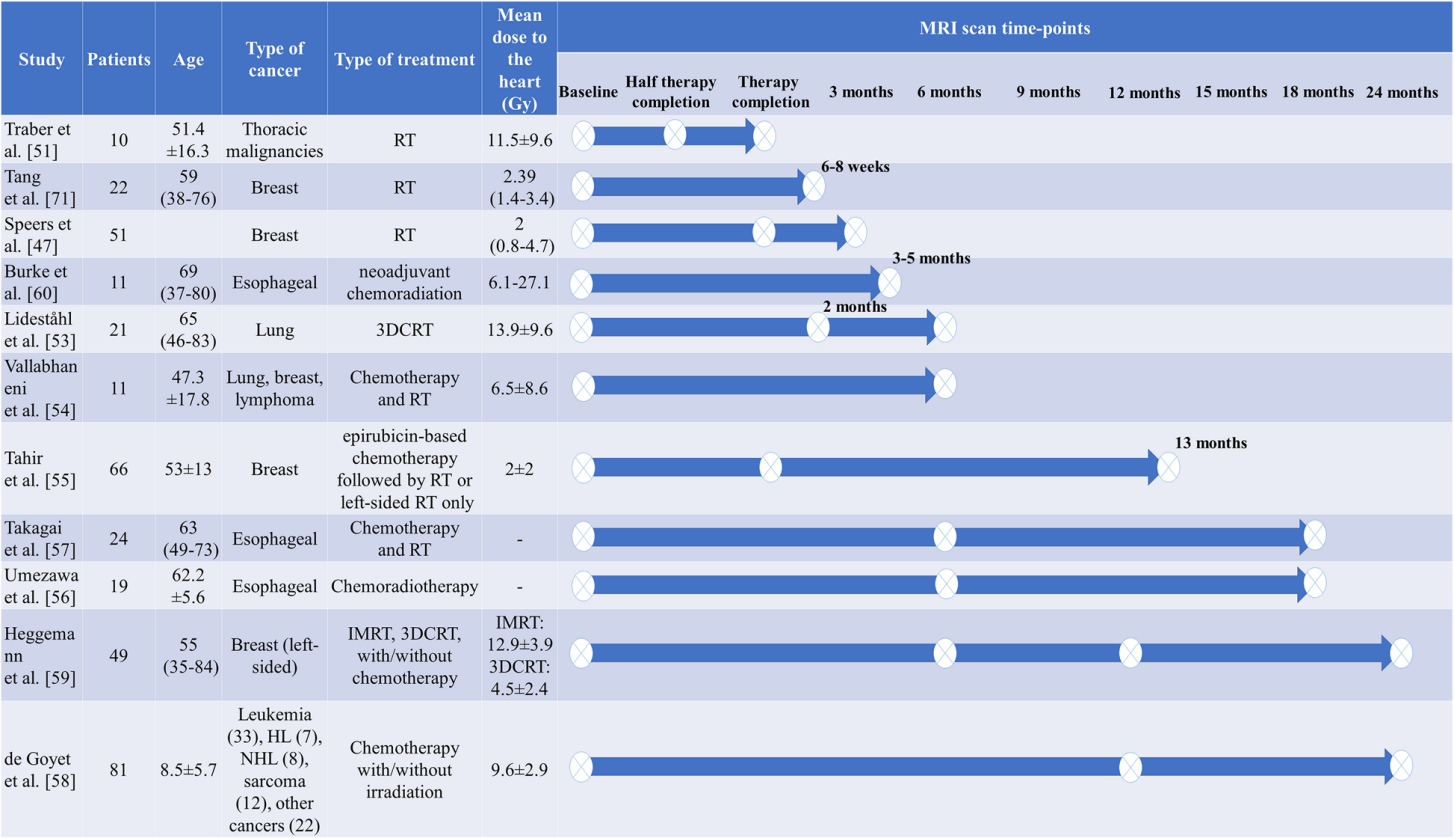
Details of longitudinal studies including number of patients, age, type of cancer, type of cancer treatment, radiation dose to the heart and timing of MRI measurements

**Fig. 3:**
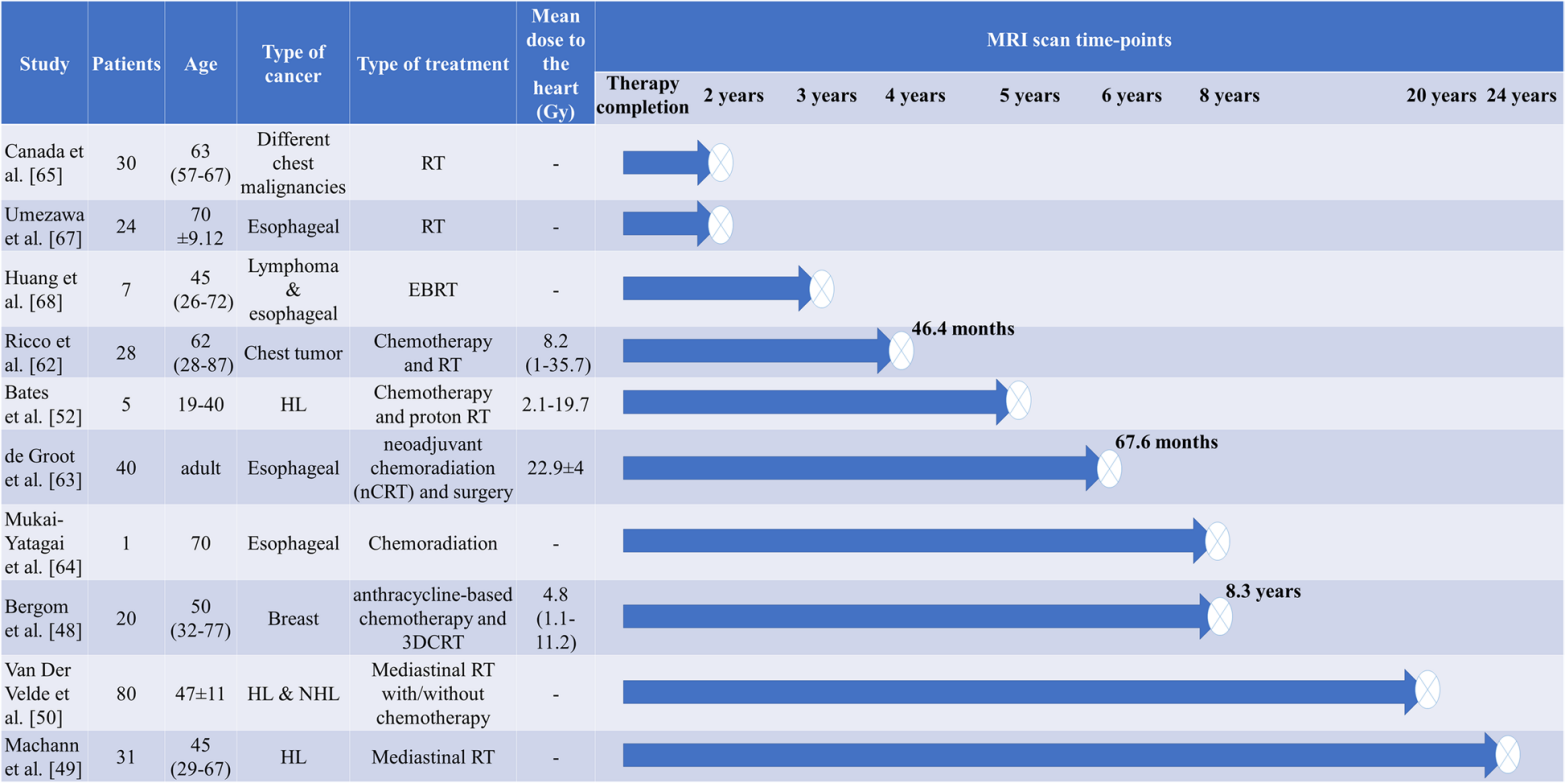
Details of cross-sectional studies including number of patients, age, type of cancer, type of cancer treatment, radiation dose to the heart and timing of MRI measurements

Figure 1 shows the workflow for identification of studies via databases following the Preferred Reporting Items for Systematic Reviews and Meta-Analyses (PRISMA) 2020 flow diagram [43]. Ten studies were cross-sectional and eleven were longitudinal. Studies included patients who received chest radiation for a number of malignancies including: breast cancer, lung cancer, esophageal cancer, and Hodgkin’s and non-Hodgkin’s lymphoma. In cross-sectional reports, the sample size, mean dose to the heart, and time of comparison since RT varied between 5-80 patients (age 19-70 years old), 2.1-22.9 Gy, and 23.5 months to 24 years, respectively. In the longitudinal studies, sample size varied from 10 to 81 patients (age 8.5-69 years old), the mean heart dose ranged between 2-13.9 Gy, and the length of serial observation (after acquiring baseline pre-RT data) ranged from 0 to 24 months after RT completion. In this review article, findings will be summarized and grouped by types of MRI-derived metrics, followed by discussion and suggestions for future works. Metrics were assessed either globally (i.e., on the whole LV or LV myocardium) or regionally (i.e., at focal locations using an American Heart Association (AHA) model or over random segmental regions). Figures 2 and 3 show a detailed summary of longitudinal and cross-sectional studies including number of patients, type of dataset (i.e., type of cancer), amount of radiation to the heart, and the timing of MRI-based measurements.

### Left ventricular ejection fraction (LVEF)

LVEF is the most common screening tool to quantify global cardiac function and is defined as the stroke volume divided by the end diastolic volume. Normal LVEF ranges between 50-70% [44]. In terms of LVEF, cardiotoxicity has been defined as any LVEF decline to <50% or an LVEF decline >10%, ≥10%, or >15% from baseline to <55%, <50%, or ≥50%, respectively [45]. Benefits of this metric and defined cutoffs for cardiotoxicity are that LVEF is a widely accepted and clinically utilized metric of cardiac function assessable by multiple imaging modalities (though these modalities may result in slightly different values for the same person [46]). A primary drawback is that declines in LVEF may not be observed in early MCT [47, 48] since global cardiac function may be able to compensate for mild early or focal cardiac damage. Thus, reliance on LVEF alone may delay the diagnosis and early treatment of MCT.

#### LVEF Decrease

A decrease in LVEF was reported in a few studies. In 31 patients with Hodgkin’s disease who were treated with mediastinal RT, LVEF dropped below 55% in 23% of patients at 24 years post-RT treatment [49]. Also, long-term survivors of Hodgkin’s lymphoma (HL) and non-HL (NHL) who were treated with mediastinal RT in combination with chemotherapy (85% of the cohort) demonstrated lower LVEF compared to healthy controls (53±5% vs 60±5%, *P<*0.001) at a median of 20 years post-treatment [50]. Finally, in a small study with ten patients with different thoracic malignancies, LVEF dropped below 50% in only one patient mid-treatment and in two patients at the end of therapy (all patients’ LVEF were above 50% at baseline) [51]. No measurements were repeated beyond the end of RT.

#### LVEF Unchanged

LVEF stayed within normal limits in multiple studies. In 20 breast cancer patients who received 3D conformal RT (3DCRT), a median LVEF of 63% was reported at 8.3 years [48]. Similar findings were noted for 5 HL survivors who underwent chemotherapy and proton RT with LVEF of 60% at 5 years post treatment [52]. Over shorter follow-ups, LVEF did not change at 3 months post-RT in 51 breast cancer patients who were exposed to a low dose of radiation to the heart (2 Gy) [47], at 2- or 6-months in 21 lung cancer patients following 3DCRT (69-70%) [53], at 6 months in 11 chest tumor patients who underwent chemoradiotherapy [54], at either therapy completion or 13 months post-therapy in 66 breast cancer patients who were treated by chemoradiotherapy (60%) or only RT (62-64%) [55], and finally at 6- or 18-months in 19 and 24 survivors of esophageal cancer, respectively, who received chemoradiotherapy (60-65%) [56, 57]. Additionally, no change in LVEF was noted for 81 pediatric cancer patients with different malignancies who were treated by chemotherapy with/without irradiation at 1-year and 2-year follow up (range: 60-62%) [58]. Finally, in 49 left-sided breast cancer patients who were treated by intensity-modulated radiation therapy (IMRT), 3DCRT, with/without chemotherapy, LVEF was measured over a 24-month follow-up. No significant reduction of LVEF was noted at 6 months in the whole cohort (59.2- 61.2%, *p=*0.059); however, patients who underwent 3DCRT with/without chemotherapy demonstrated a temporary decrease of LVEF which was resolved at 12- and 24-months. Interestingly, for the IMRT group, the LVEF increased at 24-months follow up (60.1-63.6%, *p=*0.017) [59]. Table 1 shows a summary of LVEF results for RT-induced MCT studies using MRI.

**Table 1:** LVEF changes in RT-induced MCT studies using MRI

**Study**	**Patients**	**Cancer type**	**Decrease**	**Increase**	**Constant**
*Machann* *et al.,* [49]	31	HL	<55% in 23% of patients at 24 years post-RT	-	-
*Van Der Velde* *et al.,* [50]	80	HL& NHL	53±5% vs 60±5%, *P<* 0.001 at 20 years post-RT	-	-
*Heggemann* *et al.,* [59]	49	Breast	In 3DCRT with/without chemotherapy group: temporary decrease at 6 months (59-62%, *p=*.031) In 3DCRT without chemotherapy group: temporary decrease at 6 months (59-63%, *p=*.005) resolved at 12 and 24 months: (*p=*0.443)	For IMRT group: at 24 months follow-up (60.1-63.6%, *p=*0.017)	At 6 months: the whole cohort (59.2-61.2%, *p=*0.059)
*Goyet* *et al.,* [58]	81	Leukemia, HL, NHL, Sarcoma, and others	-	-	Baseline: 62±8% One-year: 60±7% Two-year: 61±6%
*Traber* *et al.,* [51]	10	Thoracic malignancies	Half-therapy completion: (dropped below 50% in 1/10 patients) after RT completion: (dropped below 50% in 2/10 patients)	-	-
*Bergom* *et al.,* [48]	20	Breast	-	-	Normal range (63% (52-77%)) at 8.3 years post-RT
*Takagi* *et al.,* [57]	24	Esophageal	-	-	Normal range (baseline: 63±9%, 0.5 year follow up: 65±12%, 1.5 year follow up: 61±11%)
*Bates* *et al.,* [52]	5	HL	At 5 years: >5% LVEF drop in two patients with mean cardiac RT dose of ≥ 10 Gy and a total anthracycline dose of greater than 250 mg/m2	-	At 5 years: Median LVEF was 60% (52-61%)
*Umezawa* *et al.,* [56]	19	Esophagus	-	-	Normal range (baseline: 60.4±8.9%, 6 months: 62.8±12.7%, and 1.5 years: 62±10.4%)
*Vallabhaneni et al.,* [54]	11	Lung, breast, lymphoma	-	-	No significant % changes in patients with higher radiation (-10.4±7.7%) or patients with minimal radiation (-8.9±8.6%) at 6 months post-RT
*Lideståhl* *et al.,* [53]	21	Lung	-	-	Pre-RT: 69 (63-74) %, 2 months: 69.5 (65.5-74.8) %, 3 months: 70 (63-75) %
*Speers* *et al.,* [47]	51	Breast	-	-	No significant changes of LVEF at 3 months post-RT
*Tahir* *et al.,* [55]	66	Breast	-	-	In epirubicin-chemotherapy-based followed by RT group: unchanged (at baseline: 60±5%, therapy completion: 60±6% and after 13±2 months: 60±6%)
			-	-	In left-sided RT only group: unchanged (baseline: 62±5%, therapy completion: 64±6%, 13±2 months: 62±5%)

#### Discussion on LVEF

Recent RT-induced MCT studies showed no significant changes of LVEF and/or relations between LVEF and dose mostly due to short follow-ups [54, 55], low dose of radiation to the heart [47], small sample size [52], and uncertainty in the exact cumulative dose for longer follow-up studies. However, studies with longer follow-up (20 years and more) showed a decrease in LVEF [49, 50]. Also, studies that were conducted on patients treated with older RT techniques such as anterior-mantle-field, in which overdosage is expected in anterior parts of the irradiated volume, showed declines in LVEF following RT [49]. These patients with LVEF drop were treated for HL and NHL and were exposed to large RT fields covering large volumes of the mediastinum and heart with simple RT techniques. More recent studies, including those for breast cancer, have used more advanced techniques and were therefore able to reduce cardiac dose. This may also contribute to the observed lack of changes in LVEF. Overall, these findings suggest that LVEF is a poor indicator for the reliable detection of early RT-induced MCT.

### Cardiac Chamber Dimensions

A limited number of studies investigated the dimensions and mass of different cardiac chambers to find possible early changes of subclinical myocardial dysfunction and associated dose-dependency.

#### Increase in Cardiac Chamber Dimensions

Increase of cardiac volumes and dose-dependency were noted in a few studies. In 3/11 patients with esophageal cancer, LV systolic volume increased at 3-5 months following neoadjuvant chemoradiotherapy [60]. An increase of LV end-systolic volume was also noted in 80 HL and NHL patients who were treated by mediastinal RT with/without chemotherapy at 20 years compared to healthy control (*p=*0.01) [50].

Dose-dependency response was found in two studies. In 81 pediatric cancer patients (with different malignancies) who were treated with chemotherapy and irradiation, increase in indexed LV end-diastolic volume (2.1±6.5 ml/m^2^, normalized to body surface area) at 2 years was correlated with the radiation dose to 98% of the heart volume (5.8±4.6 Gy, *P<*0.05) and LV (6.1±5.0 Gy, *P<*0.05) [58]. In addition, a higher LV mass index, a predictor of cardiovascular events, was shown to be correlated with LV mean dose (*p=*0.012), V10 (LV volume that received 10 Gy, *p=*0.027), and V25 (LV volume that received 25 Gy, *p=*0.016) at a mean of 8.3 years in 20 breast cancer patients who underwent anthracycline-based chemotherapy and 3DCRT [48].

#### Cardiac Chamber Dimensions Unchanged

The dimensions of cardiac chambers stayed within normal range in two studies with breast cancer populations. In the first study with 49 patients who underwent IMRT, 3DCRT with/without chemotherapy, LV and RV volumes did not change over 24 months follow-up [59]. In the second study with 51 patients who were treated by RT, RV ejection fraction did not change at 3 months even with a temporary significant drop at the end of therapy [47].

#### Decrease in Cardiac Chamber Dimensions

Decreases of cardiac chambers were shown in two studies. At 20-year follow-up of 80 HL/NHL survivors who were treated by mediastinal RT with/without chemotherapy, a drop in RV volumes and LV diastolic volume/mass were noted (*P<*0.06) compared to healthy controls [50]. Also, in 66 breast cancer patients who were treated by epirubicin-based chemotherapy followed by RT, right- and left-sided chamber sizes were significantly decreased at the end of therapy (*P<*0.05), while only RV systolic volume and RA diastolic volume dropped significantly at 13±2 months (*P<*0.05). For those who underwent RT treatment, RV and LV volumes were reduced at both therapy completion and 13±2 months (*P<*0.05) without affecting the atrial dimensions [55]

#### Discussion on cardiac chamber dimensions

Over short follow-ups, cardiac chambers dimensions did not change and/or were recovered after therapy completion [47, 59], potentially due to compensatory features of the heart. Changes in cardiac chamber dimension were mostly noted over longer follow-up times [50], when damages are less likely to be reversible, and in concurrent treatments (e.g., epirubicin-based chemotherapy followed by RT) [55], in which cardiotoxic drugs enhance the probability of cardiac dysfunction. Finally, a relationship between radiation dose received by 98% of the volume of the heart and by the whole LV and LV end-diastolic volume was noted in pediatric cancer patients [58], who are shown to be more susceptible to radiation damage compared to adult patients [20].

### T1/T2 Mapping

T1 and T2 relaxation times are tissue-specific time-constants that can provide useful information regarding myocardial abnormalities and possible associated pathologies (e.g., fibrosis, edema) [39].

#### T1 Mapping

T1, or longitudinal relaxation time, is a biological parameter that quantifies the time required for nuclei of hydrogen atoms to recover towards thermodynamic equilibrium along the main magnetic field. It can be measured either globally or locally, which allows for quantification of heterogenous T1 distribution. T1 values differ based on local molecular environment (e.g., location-specific constitutive properties, temperature, and pressure), as well as sex, age, and other parameters. Normal native T1 values of the myocardium acquired using a modified Look-Locker inversion recovery (MOLLI) method at 1.5T and 3T scanners were reported as 950±21 ms and 1052±23 ms, respectively [61]. Notably, these values can vary with magnetic field strength, vendor, scanner model, and physical location. Pathologies can also change the tissue properties (e.g., water content) and hence the T1 values. Myocardial abnormalities associated with changes in T1 value include myocardial fibrosis, edema, inflammation, infiltrative diseases, amyloidosis, and hemosiderosis [39].

##### T1 increase

Increase of T1 signal was reported in a few studies. In 24 survivors of esophageal cancer who were treated with chemoradiotherapy, increase of T1 value was noted at 0.5 years (1257±35 ms, *P <*0.01) and 1.5 years (1238±56 ms, *P<*0.024) compared to the baseline (1183±46 ms) at the basal septum (a highly irradiated area (43±4 Gy)). However, no correlation was found between regional radiation dose and percent change of T1 at the basal septum [57]. Over longer follow-up periods, a significant increase of T1 was found in 80 HL and NHL survivors at 20 years following mediastinal RT with/without chemotherapy compared to healthy controls (980±33 ms vs 964±25 ms, *p=*0.01) [50].

##### T1 unchanged

The majority of studies found T1 values within normal range. During RT of 10 patients with different thoracic malignancies, no T1 signal changes were noted at half-therapy (956±14 ms) and at therapy completion (968±72 ms), compared to baseline (966±39 ms) [51]. A similar cohort of patients in another study demonstrated no T1 changes at 6 months in either highly or minimally irradiated patients [54]. Over longer follow-up periods, T1 in 28 patients with chest tumors stayed around 1009 ms (*p=*0.054) with no dose-dependent response at 46.6 months following chemoradiotherapy [62]. T1 signal also remained unchanged among 40 esophageal cancer patients who underwent neoadjuvant chemoradiotherapy (959.2±34.7 ms) compared to control (949.9±28.4 ms) (*p=*0.4) at 67.6 months [63].

Fluctuation of T1 values over different follow-up times was noted in a single study of 66 patients with breast cancer who were treated by epirubicin-based chemotherapy followed by RT or with left-sided RT alone. T1 value increased at therapy completion in the group receiving epirubicin-based chemotherapy followed by RT and returned to baseline at 13±2 months, while the group receiving left-sided RT only demonstrated no significant changes in T1 at both follow-ups [55].

##### T1 decrease

Decrease of T1 signal was found in 51 patients with breast cancer post-RT treatment (immediately at the end of treatment, -20 ms, *p=*0.022 and at 3 months, -23 ms, *P<*0.001) [47]. A summary of T1 value findings using MRI for RT-induced MCT studies is shown in Table 2.

**Table 2:** T1 value changes in RT-induced MCT studies using MRI (no dose-dependency was reported)

**Study**	**Patients**	**Cancer type**	**Decrease**	**Increase**	**Constant**
*Van Der Velde et al.,* [50]	80	HL & NHL	-	Significant increase of native T1 values compared to healthy control at 20 years post-RT (980±33 ms vs 964±25 ms, *p=*0.01)	-
*Traber et al.,* [51]	10	Thoracis malignancies	-	-	Normal range: (baseline (966±39 ms), half-time RT (956±14 ms), and after RT (968±72 ms))
*Takagi et al.,* [57]	24	Esophagus	-	In the basal septum (highly radiated area, 43±4 Gy), native T1 values were higher at 0.5 year (1257±35 ms, *P<*0.01) and 1.5 year (1238±56 ms, *P<* 0.024) compared to the baseline (1183±46 ms)	At the apical lateral wall (nonradiated area 3±4 Gy), no significant T1 differences were found at different time points
*Speers et al.,* [47]	51	Breast	End of the treatment (−20 ms, *p=*0.022) and three months post-treatment (−23 ms, *P<* 0.001)	-	-
*Tahir et al.,* [55]	66	Breast	-	In epirubicin-chemotherapy-based followed by RT group: baseline: 1244±29 ms, therapy completion: 1293±34 ms, *P<*0.001)	In epirubicin-chemotherapy-based followed by RT group: changes returned to baseline at 13±2 months (1250±26 ms)
			-	-	In left -sided RT only group: constant (baseline: 1237±29 ms, at therapy completion: 1237±42 ms, and 13±2 months: 1239±39 ms)
*Ricco et al.,* [62]	28	Chest tumor	-	-	Mean T1: 1009 ms (range 933–1117 ms) with no dose-dependency at 46.4 months post-RT (*p=*0.054)
*Vallabhaneni et al.,*[54]	11	Lung, breast, lymphoma	-	-	No significant %T1 changes in patients with higher radiation (-1.3±3.7%) or patients with minimal radiation (-3.7±2.0%) at 6 months post-RT
*de Groot et al.,* [63]	40	Esophagus	-	-	No differences between neoadjuvant chemoradiotherapy and control (959.2±34.7 ms vs 949.9±28.4 ms, *p=*0.4) at 67.6 months

##### Discussion on T1 mapping

Different behaviors of T1 signal (e.g., increase, decrease, unchanged) were reported following cancer treatment. Increase of T1 was noted in longer follow-ups [50], regional analysis at high-dose regions [57], and with concurrent treatments [55]. Another explanation is the type of cancer. For example, in breast cancer patients, the mean heart dose is low; typically, only a small region of the LV is irradiated. Therefore, it is not surprising that there are no T1 changes [55]; whereas in patients with esophageal cancer, much larger volumes of the heart can be in the high-dose fields [57]. Despite increase of T1 value in high dose regions, no correlations were found between T1 increase and dose [50, 57]. Unchanged T1 values were mostly seen in heterogenous patient population [62], shorter follow-ups, [51] and non-irradiated regions [57]. Interestingly, one study reported a decrease of T1 at the end of treatment and three months post-treatment [47]. Given these findings, it might be premature to conclude the consistent progression of fibrosis and/or inflammation shortly after RT in all cases. Further studies are required to examine this matter more fully.

#### T2 Mapping

Similar to T1, T2 (or transverse relaxation time) is a biological parameter that is tissue-specific. Normal T2 values of myocardium acquired from steady-state free precession (SSFP) technique ranged between 52.18±3.4 ms and 45.1 ms at 1.5T and 3T scanners, respectively. T2 changes are mostly associated with change of water content in the tissue. The main pathology associated with longer T2 values is myocardial edema [39].

##### T2 unchanged

In RT-involved treatments, T2 values were reported to be within normal range. At 24 years follow up of 80 HL and NHL survivors who were treated with mediastinal RT with/without chemotherapy, T2 values did not differ from healthy controls (both 50 ms, *p=*0.13) [50]. In shorter follow-ups, T2 signal in 51 breast cancer patients did not change at therapy completion or 3 months post-RT [47]. Similar findings were noted in highly or minimally irradiated patients with chest tumors at 6 months post chemoradiotherapy [54].

In 66 breast cancer patients with two different treatments, T2 stayed unchanged in the left-sided RT group at the end of therapy and 13±2 months post-RT (46-47 ms); however, in patients who underwent epirubicin-based chemotherapy followed by RT , T2 increased at therapy completion (48 ms vs 45 ms, *P<*0.001) and returned to baseline at 13±2 months (46 ms) [55]. Table 3 shows T2 signal changes in RT-induced MCT reports using MRI.

**Table 3:** T2 signal changes in RT-induced MCT studies using MRI (No decrease or dose-dependency were reported)

**Study**	**Patients**	**Cancer type**	**Increase**	**Constant**
*Van Der Velde et al.,* [50]	80	HL & NHL	-	No differences were noted between cancer patients and healthy control at 20 years post-RT (50±3 ms vs 50±2 ms, *p=*0.13)
*Speers et al.,* [47]	51	Breast	-	No significant changes of T2 signal over different time points (therapy completion and 3 months post-RT)
*Vallabhaneni et al.,* [54]	11	Lung, breast, lymphoma	-	No significant %T2 changes in patients with higher radiation (3.9±9.5%) or patients with minimal radiation (-3.4±12.1%) at 6-months following RT
*Tahir et al.,* [55]	66	Breast	In epirubicin-chemotherapy-based followed by RT group: increased at therapy completion compared to baseline (48±3 ms vs 45±3 ms, *P<*0.001)	In epirubicin-chemotherapy-based followed by RT group: returned to baseline at 13±2 months (46±3 ms)
			-	In left-sided RT only group: constant at baseline (46±3 ms), therapy completion (47±2 ms) and after 13±2 months (46±3 ms)

##### Discussion on T2 mapping

T2 signal stayed unchanged over longer follow-ups [50] (with a higher likelihood of progression of fibrosis rather than edema) and over shorter follow-ups at low dose (e.g., 2 Gy) [47], except for concurrent treatments [55]. Even with concurrent treatments, the changes were resolved in a few months. These findings can demonstrate the low likelihood of myocardial edema development following RT [47, 50, 55]. Regional analysis of T2 mapping in longitudinal studies of patients with higher dose to the heart over both short and long-term follow ups are required for further evaluation.

### Extracellular volume fraction (ECV)

The cellular components of myocardium, including the interconnected cardiac muscles, are embedded in a complex three-dimensional extracellular space that accounts for the interstitial (or extracellular) component of the myocardium. One of the distinct features of myocardial pathologies (e.g., myocardial fibrosis, inflammation, edema) is the expansion of this extracellular space. Quantitative evaluation of extracellular expansion is now possible by acquisition of the hematocrit and pre- and post-T1 values of myocardium and blood pool (before vs after administration of a contrast agent (e.g., gadolinium)). Equation 1 shows the formula for ECV calculation.

1 $$ECV=\left(1-hematocrit\right)\times \frac{(\frac{1}{{T1}_{myo post}}-\frac{1}{{T1}_{myo pre}})}{(\frac{1}{{T1}_{blood post}}-\frac{1}{{T1}_{blood pre}})}$$

Normal myocardial ECV values of 25±4% and 26±4% at 1.5T and 3T, respectively, have been reported [39, 61]. It should be noted that ECV values even in healthy volunteers may differ based on age, sex and type of scanner [39].

#### ECV increase

Increase of ECV and its dose-dependency have been reported in a few recent studies. Increase of ECV (32% vs 26%, *P<*0.01) was noted at 6 months post chemoradiotherapy in 24 patients with esophageal cancer in high dose regions (43±4 Gy) [57]. Segmental analysis of ECV (i.e., measured over focal regions) in 40 patients with esophageal cancer who received neoadjuvant chemoradiotherapy showed a linear relationship between mean dose per segment and ECV (a 0.136%-point increase of ECV for each Gy (*P<*0.001)) at 67.6 months [63]. Lastly, a substantial increase of ECV (45%) was also reported in an esophageal cancer patient at 8 years following chemoradiotherapy [64].

#### ECV unchanged

No significant change and/or relation between ECV and dose were also reported in a few studies. In 80 HL and NHL survivors who were treated by mediastinal RT with/without chemotherapy, ECV did not differ compared to healthy controls in patients with 20-year follow-up (28% vs 29%, *p=*0.24) [50]. Similarly, global ECV (i.e., in the whole myocardium) of 27% was measured in the myocardium of 20 breast cancer patients after 8.3 years of anthracycline-based chemotherapy and 3DCRT [48]. Over shorter time-points, ECV stayed unchanged at 2 years post-RT for 30 patients with various chest malignancies [65] and among 66 breast cancer patients who underwent epirubicin-based chemotherapy followed by RT (28% vs 29%) or left-sided RT treatment only (30% vs 30%) at 13±2 months follow-up [55]. A 6-months follow-up of 11 patients with various chest tumors also did not show significant ECV changes between highly or minimally irradiated patients [54]. Table 4 shows ECV measurements following RT treatment.

**Table 4:** ECV changes in RT-induced MCT studies using MRI (No decrease of ECV was reported)

**Study**	**Patients**	**Cancer type**	**Increase**	**Constant**	**Dose dependent**
*Van Der Velde et al.,*[50]	80	HL& NHL	-	No differences with healthy control at 20 years post-RT (28±3% vs 29±3%, *p=*0.24)	-
*Bergom et al.,* [48]	20	Breast	-	Mean global ECV of 27% (range: 23-34%) at 8.3 years post-RT	-
*Takagi et al.,* [57]	24	Esophagus	In basal septum (high radiated area, 43±4 Gy) in 0.5 year follow up (26±3% vs 32±3%, *P<*0.01)	-	-
*Tahir et al.,* [55]	66	Breast	-	In epirubicin-chemotherapy-based followed by RT group: (Baseline: 28±2% and 13±2 months: 29±2%, *p=*0.52)	-
			-	In left-seded RT only group: (baseline: 30±3% and 13±2 months: 30±3%)	-
*de Groot et al.,* [63]	40	Esophagus	(28.4±1.0% vs 24.0±0.9%; *P<*0.001)	-	Linear relation between mean dose per segment and ECV increase (a 0.136%-point increase of ECV for each Gy (*P<*0.001) at 67.6 months
*Canada et al.,* [65]	30	Chest malignancies		no associations with dose: median 28% [26–31] at 2 years post-RT	-
*Mukai-Yatagai et al.,* [64]	1	Esophagus	Increase of ECV (45%) at 8 years	-	-
*Vallabhaneni et al.,* [54]	11	Lung, breast, lymphoma	-	No significant % ECV changes in patients with higher radiation (-11.5±20.8%) or patients with minimal radiation (-8.1±2.9%) at 6-months following RT	-

### Discussion on ECV

Global analysis of ECV [50] and short follow-up times (less than 2 years) [55, 65] did not show any changes post-RT. However, segmental analysis (i.e., by the American Heart Association (AHA) model) of ECV at high-dose regions over short follow-up times (0.5 year) [57] and global analysis over longer follow-ups in concurrent treatments (greater than 67.6 months) [63] demonstrated an increase of ECV signal. This might suggest that short follow-up increases in ECV are associated with focal and/or short-term inflammation caused by chemoradiotherapy, followed by fibrosis. Though, it should be noted that in concurrent treatments, such as the use of cisplatin (which increases the risk of late cardiovascular events) and 5-fluorouracil (which is associated with myocardial ischemia), it may be difficult to differentiate the effects on the interstitial myocardium (i.e., ECV changes) from RT alone versus systemic treatment [57]. ECV was also elevated at higher RT dose regions [57]. More importantly, one study found a linear relationship between ECV and segmental mean dose as a 0.136%-point increase of ECV for each Gy (*P <*.001) [63]. Further systematic analysis of ECV is necessary to determine its value for identifying fibrosis and/or inflammation associated with RT-induced MCT. It should also be noted that ECV, like T1/T2 but in lesser extent, is subject to scanner and patient-specific variabilities; therefore, longitudinal studies where each follow-up measurement is compared to its baseline value are more meaningful to report.

### Late Gadolinium enhancement (LGE)

LGE MRI is a standard non-invasive method for assessing ischemic and nonischemic myopathic processes with high spatial resolution [66]. Notably, LGE can detect increase of extracellular space that represents fibrous scar tissue [62]. No enhanced area (0% LGE signal/volume) is expected for healthy tissues, while increases in LGE are expected with increased degree of scaring.

#### LGE Increase

Enhanced LGE volumes were reported in a few studies. In a study of patients with Hodgkin’s disease survival of at least 20 years, 29% of 31 patients demonstrated late enhancement 24 years following mediastinal RT [49]. Similarly, LGE was noted at 20 years in 25% of 80 HL and NHL survivors who were treated by mediastinal RT with/without chemotherapy [50]. In another study of patients with various chest tumors, 9 of 28 patients showed LGE (2.3 ml (0.2-6.1)) at 46.4 months post-chemoradiotherapy [62]. Over shorter follow-ups, the prevalence of LGE was increased at 1.5 years following chemoradiotherapy compared to baseline (78% vs 7%, *P<*0.01) among 24 esophageal cancer patients [57]. Also, in 11 esophageal patients, LGE was noted in the subepicardial and mid-wall portion of the myocardium at 3-5 months after neoadjuvant chemoradiotherapy [60].

A few studies showed dose-dependent LGE response. In 12 out of 24 esophageal patients, LGE was detected in 15.38% of AHA segments receiving 40 Gy and in 21.2% of AHA segments receiving 60 Gy at 23.5 months post-RT [67]. In another study, a progressive increase of LGE signal was noted at >30 Gy dose in 68% (13/19) of esophageal cancer patients at 6 months and 1.5 year following post-chemoradiotherapy [56]. Also, in two years follow-up of children who were undergoing chemotherapy with/without RT for various malignancies, increase of LV myocardial scaring (0.4±1.5%, *P<*0.05) was correlated with the dose to 20% of LV volume (11.9±4.0 Gy, *P<*0.05) [58].

Lastly, one study was done on evaluation of RT-induced LA chamber enhancement at 3.1 years among 7 patients with lymphoma and esophageal cancer who were treated with RT. They found that there is a linear relationship between the LA mean dose (25.9 Gy) and its scar volume (2.5 cm3) (*p=*0.03) and between the ratio of LA scar-to-wall volume and dose (*P<*0.01) at 3.1 years [68].

#### LGE unchanged

On the other hand, no signs of LGE were found in 10 patients with different thoracic malignancies during RT treatment [51], 21 lung cancer patients at 2- and 6-months post-3DCRT [53], 66 patients with breast cancer (at 13 months) who were treated by either epirubicin-based chemotherapy followed by RT or left-sided RT only [55], 20 breast cancer patients at 8.3 years after anthracycline-based chemotherapy and 3DCRT [48], 40 esophageal cancer patients at 67.6 months past neoadjuvant chemoradiotherapy [63], or a 70-year-old esophageal cancer patient who was examined at 8 years post-chemoradiotherapy [64].

#### LGE decrease

Decreases in LGE signal were noted in low dose regions at 0.5 year (-0.2% change in 0-10 Gy regions) and 1.5 year (-0.8%, -3.2%, -1.9%, -4.4% changes corresponding to 0-10 Gy, 10-20 Gy, 20-30 Gy, 30-40 Gy of radiation, respectively) post chemoradiotherapy in 19 patients with esophageal cancer [56]. A summary of LGE findings in RT-induced MCT analyses is reported in Table 5.

**Table 5:** LGE changes at RT-induced MCT studies using MRI

**Study**	**Patients**	**Cancer type**	**Decrease**	**Increase**	**Constant**	**Dose dependent**
*Machann et al.,*[49]	31	HL	-	26% and 3% of the whole cohort demonstrated ischemic and cardiomyopathic late enhancement, respectively at 24 years post-RT	-	-
*Van Der Velde et al.,* [50]	80	HL & NHL	-	In 25% of patients at 20 years post-RT.	-	-
*Traber et al.,* [51]	10	Thoracic malignancy	-	-	Only 2 patients (out of 10) demonstrated LGE at the baseline with no other LGE findings during RT	-
*Bergom et al.,*[48]	20	Breast	-	-	No LGE was observed at 8.3 years.	-
*Takagi et al.,* [57]	24	Esophagus	-	LGE changes were noticed at 1.5 year follow up among 79% of participants (7% vs 78%, *P<*0.01)	No significant prevalence of LGE at 0.5 year follow up (*p=*0.16)	-
*Tahir et al.,* [55]	66	Breast	-	-	No LGE at all timepoints for both treatments	-
*Umezawa et al.,*[67]	24	Esophagus	-	Higher LGE percentage in 60 Gy dose line (21.21%), and 40 Gy dose line (15.38%) in 12 of 24 patients who demonstrated LGE increased in 23.5 months	0% LGE percentage at segments out of the radiation field	-
*Umezawa et al.,*[56]	19	Esophagus	At 6 months: -0.2% signal decrease corresponding to 0-10 Gy At 1.5 year: -0.8%, -3.2%, -1.9%, -4.4% signal decrease corresponding to 0-10 Gy, 10-20 Gy, 20-30 Gy, 30-40 Gy, respectively.	At 6 months: 0.4%, 1.1%, 5.7%, 35.7%, and 38.1% signal increase corresponding to 10-20 Gy, 20-30 Gy, 30-40 Gy, 40-50 Gy, and 50-60 Gy, respectively. At 1.5 year: 17.5%, and 20.1% signal increase corresponding to 40-50 Gy, and 50-60 Gy, respectively.	-	A progressive increase of signal was noted at >30 Gy at both time points (6 months and 1.5 year).
*Huang et al.,* [68]	7	Lymphoma & esophagus	-	-	-	1) A linear relation between LA scar-enhanced volume and mean dose with an average LA scar volume of 2.5 cm^3^ and average mean dose of 25.9 Gy (R2 = 0.8514, *p=*0.03) at 3.1 years. 2) linear relation between radiation received by the cardiac tissue and the ratio of (LA scar/LA wall) at 3.1 years.
*Burke et al.,* [60]	11	Esophagus	-	LGE was noted at the subepicardial and mid-wall after 3-5 months following therapy	-	-
*Mukai-Yatagai et al.,* [64]	1	Esophagus	-	-	No signs of LGE at 8 years	-
*Ricco et al.,* [62]	28	Chest tumor	-	LGE noted in 9/28 patients of 2.3 ml (0.2-6.1), at left myocardium or septum at 46.4 months.		-
*de Groot et al.,*[63]	40	Esophagus	-	-	No significant difference between neoadjuvant chemoradiotherapy and control group at 67.6 months post-RT	-
*Lideståhl et al.,*[53]	21	Lung	-	-	No visual signs of LGE were detected at 2- and 6-month post-RT	-
*de Goyet et al.,*[58]	81	Leukemia, HL, NHL, sarcoma, and other cancers	-	At 2-year follow-up (4.4±2%) compared to baseline (4.0±1.6%), *P<*0.05	Between baseline (4.0±1.6%) and 1-year follow-up (4.3±2)	Increase of LV myocardial scarring (0.4±1.5%, *P<*0.05) at 2-year follow-up was correlated with the radiation dose received by 20% of volume of the LV (11.9±4.0 Gy; r=0.85; *P<*0.05).

### Discussion on LGE

Global changes of LGE were mostly noted over longer follow-ups (>20 years) [50], particularly in patients treated by older RT treatment techniques (e.g., mediastinal RT with anterior mantle-field technique) [49], and/or those who received higher dose to the heart (>22.9±4 Gy) [63]. Over shorter follow-ups (3-5 months), only those with segmental analysis showed an elevated LGE signal after RT [57, 60, 67]. Dose-dependent response of LGE was also noted in a few studies. A linear relationship was found between LA enhanced LGE volume and average received dose (25.9 Gy) [68]. Similarly, a progressive increase of LGE signal was found at >30 Gy (threshold) [56] with higher signal intensity changes at shorter follow-ups (6 months vs 1.5 year), suggesting that RT-induced inflammatory response may be present for 6 months and diminish in 1.5 years [56]. Finally, enhanced volumes on LGE MRI were noted in younger patients. Children, as a high risk group, showed a dose-dependent increase of LV myocardial scarring which could be due to myocardial hypoperfusion or an early sign of radiation damage [58].

Several studies did not find any signs of LGE, potentially due to small sample size (e.g., 1 patient) [64], low radiation dose to the heart (e.g., 2±2 Gy) [55], global (rather than regional) analysis [48], and non-ideal timing of follow-up (i.e., too early to detect acute inflammation/edema or too late to detect the formation of scar tissue as a late radiation effect (2-6 months follow-up)) [53]. Nevertheless, dose-dependency of LGE and ECV findings indicate the potential of CMR to detect early changes/correlations between MCT (possibly myocardial fibrosis) and RT dose.

### Strain (circumferential, radial, longitudinal)

Cardiac function is a combination of contraction, twist, and expansion of myocardium in multiple axes. Regional and global quantification of myocardial deformation (e.g., via measurement of strain) in any of these axes may serve as a potential metric to detect sub-clinical myocardial changes and assess myocardial function. Strain can quantify spatial components of contractile function over multiple directions (e.g., radial, circumferential, and longitudinal). With regards to the LV, MRI-derived strain measurements in healthy subjects have reported pooled mean values of global longitudinal strain (GLS), global circumferential strain (GCS), and global radial strain (GRS) as -18.6% (-19.5% to -17.6%), -21.0% (−22.4% to -19.6%), and 38.7% (30.5% to 46.9%), respectively [69]. Notably, a relative drop of greater than 15% of GLS is known to be clinically significant and could be an early indicator of CVT [28, 70].

#### Regional (decrease)

In 22 breast cancer patients who underwent RT, decrease in the magnitude of circumferential, radial, and longitudinal strains were noted in segmental regions 6 weeks post-treatment, with a negative correlation between radial strain and maximum dose in segments 6 and 14 (*P<*0.05) of the 16 segment AHA model, as well as a negative correlation between the magnitude of longitudinal strain and dose in segment 6 (*P<*0.01) [71] (Note that longitudinal and circumferential strain are typically negative when using diastole as the reference configuration).

#### Global (decrease/constant)

Global measurements of strain were reported in a few studies. In longer follow-ups, significant decreases in the magnitude of GLS, GRS, and GCS were reported in 80 HL and NHL survivors 20 years following mediastinal RT with/without chemotherapy [50]. Similarly, a drop in GLS magnitude (-14.6%) was shown after 8.3 years among 16 of 20 breast cancer patients who were treated with anthracycline-based chemotherapy and 3DCRT [48]. Over shorter follow-ups, GLS and GCS were reduced in magnitude in breast cancer patients with concurrent treatment (epirubicin-based chemotherapy followed by RT) at both therapy completion and 13±2 months post therapy (-18% to -17% for both strains and timepoints) [55]. On the other hand, 6-months follow up of 11 patients with various chest tumors did not show any significant strain changes [54]. Also, no relation was found between global strain and dose in any of the studies. Table 6 summarizes strain changes in RT-induced MCT studies using MRI.

**Table 6:** Strain changes in RT-induced MCT studies using MRI (No increase of strain was reported)

**Study**	**Patients**	**Cancer type**	**Decrease**	**Constant**	**Dose-dependent**
*Van Der Velde et al.,* [50]	80	HL & NHL	GLS (-19.5±2.5 vs -20.6±2, *p=*0.01) GCS (-17.9±2.5 vs -20.4±2.2, *P<*0.001) GRS (69±15 vs 76±15, *p=*0.02) at 20 years post-RT.	-	-
*Vallabhaneni et al.,* [54]	11	Lung, breast, lymphoma	-	GLS: No significant % changes in patients with higher radiation (-15.2±15.2%) or patients with minimal radiation (-6.8±3.1%) GCS: No significant % changes in patients with higher radiation (-8.4±14.5%) or patients with minimal radiation (-4.0±6.9%) at 6-months following RT.	-
*Bergom et al.,* [48]	20	Breast	Abnormal lower absolute strain values in 16/20 patients -14.6% (-17.8% to -11.1%) compared to normal range -22.1% to -15.9% at 8.3 years post-RT	-	-
*Tahir et al.,* [55]	66	Breast	In epirubicin-chemotherapy-based followed by RT group: GLS (baseline: -18±2%, therapy completion: -17±2%, *p=*0.01; 13±2 months: -17±2% *p=*0.01) GCS (baseline: -18±2%, therapy completion: -17±3%, *p=*0.03; 13±2 months: -17±3% *p=*0.01)	In epirubicin-chemotherapy-based followed by RT group: GRS changes were not significant (baseline: 36±7%; post-therapy completion: 34±8%; 13±2 months post-therapy completion: 34±6%)	-
			-	In left-sided RT group: GLS (baseline: -18±2%, therapy completion: -18±2%, 13±2 months post-therapy completion: -18±1%) GCS (baseline: -18±2%, therapy completion: -18±2%, 13±2 months post-therapy completion: -19±3%) GRS (baseline: 39±6%, therapy completion: 39±9%, 13±2 months post-therapy completion: 39±7%)	-
*Tang et al.,* [71]	22	Breast	At 6 weeks vs pre-treatment: 3D circumferential: segment 7 -16.39 vs -19.17, segment 8 -18.73 vs -20.67, segment 13 -15.95 vs -18.48. 2D circumferential: segment 1 -19.13 vs -23.57, segment 14 -25.01 vs -28.29. 3D radial: segment 8 25.46 vs 35.32, segment 9 17.54 vs 27.24. 3D longitudinal: segment 7 -19.12 vs -21.44	-	Negative correlation: 3D radial at segment 6 and max dose (-0.443, *p=*0.05), 2D radial at segment 14 and max dose (-0.543, *p=*0.01), Positive correlation: 3D longitudinal strain at segment 6 and max dose (0.669, *P<*0.01).

### Discussion on strain

Similar to other global metrics (e.g., LVEF), no correlation was found between GLS/GCS/GRS and dose [48, 50, 55]. However, segmental strain analysis showed a potential relationship between local strain reduction and radiation dose [71]. Significant reduction of strain (without dose-dependency) was noted in longer follow-up studies [48, 50]. It is unclear if this reduction is due solely to time or whether concurrent treatments (e.g., anthracycline and RT exposure) and/or other cardiac risk factors play a significant role. Longitudinal studies over longer and shorter follow-up times in patients with single and concurrent treatments are needed to fully examine this matter. Lastly, it has been shown that strain can detect myocardial changes earlier than other global metrics (e.g., LVEF) [72]. For example, compensatory features of the heart can preserve LVEF by increasing cardiac torsion following a drop in circumferential strain due to myocardial fiber dysfunction [28, 73]. Therefore, it may be beneficial to utilize strain measurements over LVEF alone if one aims to measure global cardiac function following RT treatment.

## Image Registration

### Clinical importance

To evaluate the effects of radiation to the heart and its substructures, it is important to accurately align the planning CT (where the spatially heterogeneous clinical dosimetry map is calculated) with magnetic resonance images that are more sensitive in detecting MCT across different time points, patient positions, and respiratory and cardiac phases. Without accurate registration of the dose and the MRI, accurate analyses of dose-effect correlations are not feasible. Lacking quantitative registrations, the uncertainties associated with simple visual comparisons or simplistic registrations between dose and MRI likely contribute to the overall variation between different studies, time points, tumors, and measurement techniques of the relevant parameters.

**Fig. 4:**
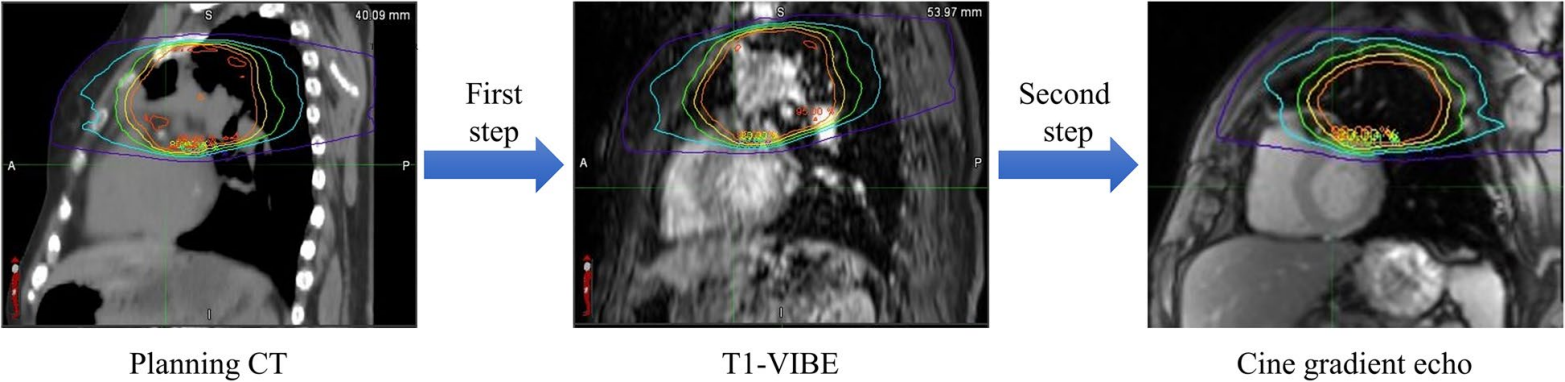
Workflow of transferring a dose map from planning CT onto a 2D MRI sequence (e.g., cine gradient echo) using rigid/deformable image registration. T1-VIBE as a 3D MRI sequence is necessary to compensate for the limited field of view between 3D CT and 2D MR images

Recent studies have shown that mono- and multi-modality image registration of the heart, LV and thoracic aorta on CT and MR datasets in the axial plane at various time points, breathing phases, and contrast levels can comply with American Association of Physicists in Medicine (AAPM) Task Group (TG) 132 criteria (mean distance to agreement (MDA) < 3 mm and Dice > 0.8) [74]. It should be noted that direct registration of 3D planning CT onto 2D MR images (e.g., cine gradient echo) cannot be directly accomplished due to the limited field of view and insufficient nearby anatomical landmarks in 2D MR images, which hampers the ability to localize them reliably onto 3D CT. A feasible two-step workflow has been suggested [75, 76] with an initial registration between the planning CT and a 3D MRI sequence (e.g., T1-volumetric interpolated breath-hold examination (VIBE)) and a secondary registration between T1-VIBE and 2D MR images, as illustrated in Fig. 4.

### Image registration algorithm

Image registration is a transformation process in which the features from one coordinate space are mapped onto another space. One image is considered as reference (static) while the other images move/deform based on the reference image with the following strategic approaches: spatial transformation (e.g., rigid/non-rigid, 2D to 2D, 3D to 3D, 2D to 3D), interpolation (intensity-based or object-based), similarity measurements (point-based, surface-based, intensity-based), and finally optimization (iterative parameters used to converge the similarity measurements to the optimal value). Proper selection of the image registration workflow depends on the modalities, dimensions, and degree of deformation. Accurate image registration can provide complementary information from two different datasets to improve therapeutic decision-making and to examine spatially registered cross-modality correlations [77–79].

### Dose and CMR-findings using image registration

Despite the importance of image registration, particularly in the quantitative mapping of CT-derived RT dose onto MRI datasets, few studies have incorporated image registration in their work [60]. As a result, the correlation of dose and patient-specific changes in regional MRI-derived metrics often have been limited to simple visual comparisons of dose measured on the planning CT to metrics at estimated corresponding locations on unregistered MRI datasets [63]. However, there have been a few studies that did include image registration in their workflow. For example, in an evaluation of RT-induced myocardial damage in esophageal cancer patients treated with chemoradiotherapy, planning CT and pre-treatment LGE MRI were rigidly registered, while the pre-treatment LGE MRI and post-treatment LGE MRI underwent multimodal deformable image registration. This approach allowed the calculation of a dose-response curve for LGE [56]. In another LGE MRI analysis of late radiation damage on LA fibrosis in patients with previous exposure to external beam RT, 3D CT and 3D LGE MR images were rigidly registered using auto-matching (i.e., the process of extraction and matching similar features from pairs of images) with mutual information processing followed by manual translation/rotation for registration enhancement at the regions of interest [68]. Similarly, CMR and planning CT fusion was performed using 3D rigid registration of the LV by auto-matching followed by manual adjustments to explore RT-induced heart disease using LGE MRI [62].

## Discussion

MRI is considered a ‘gold standard’ modality for left ventricular volume quantification [80] and has shown great potential for detecting regional and global dysfunction and other abnormalities following cancer therapy. Early detection of RT-induced MCT using MRI techniques may increase the opportunity for clinical diagnosis and timely intervention to prevent irreversible damage.

Global metrics (e.g., LVEF) have been shown to detect myocardial changes at longer follow-ups (>20 years) [50] when damage is frequently permanent and/or in patients who have been treated with older RT techniques and large fields (e.g., anterior mantle-field) [49] that deliver high doses to large heart volumes. Changes in other global metrics, such as global strain, have been shown to precede LVEF changes [72], with the earliest changes being noted over shorter follow-ups (e.g., 13±2 months) in patients undergoing concurrent treatments (e.g., chemoradiotherapy) [55], in which the cardiotoxic effects of chemotherapy and radiotherapy may be compounded. In this review of RT effects assessed with MRI, no dose-dependency was observed in global measurements, except for changes in cardiac chamber dimensions in either pediatric patients (e.g., LV diastolic volume correlated with heart/LV dose) over short follow-ups (<2 years) [58], who are more susceptible to radiation damage [20], or in patients undergoing concurrent treatment over longer follow-ups (e.g., LV mass index correlation with LV mean dose at >8.3 years) [48].

Regional metrics have been shown to have more potentials to detect early changes and/or dose-dependencies. For example, segmental strain analysis over short follow-ups (e.g., 6-8 weeks) has shown a dose-dependent response [71] since high radiation doses may alter the myocardial contractibility in response to either direct local radiation damage or potentially as compensation for RT-induced damage to other parts of the heart or vasculature. In addition, relationships between regional MRI-based metrics and dose, such as changes in T1 signal over high dose regions [57], have been demonstrated. Notably, a segmental ECV analysis manifested a 0.136% average increase of ECV per Gray of mean segmental dose [63]. In addition, LV myocardial scarring has been correlated with LV mean dose [58], and >30 Gy of radiation has been identified as a threshold at which a progressive increase of LGE is detected [56]. These findings are mostly associated with heterogeneous dose distributions of RT and a higher probability of toxicity occurring in regions with elevated doses, which emphasizes the importance of regional myocardial assessments using novel MRI techniques.

**Fig. 5:**
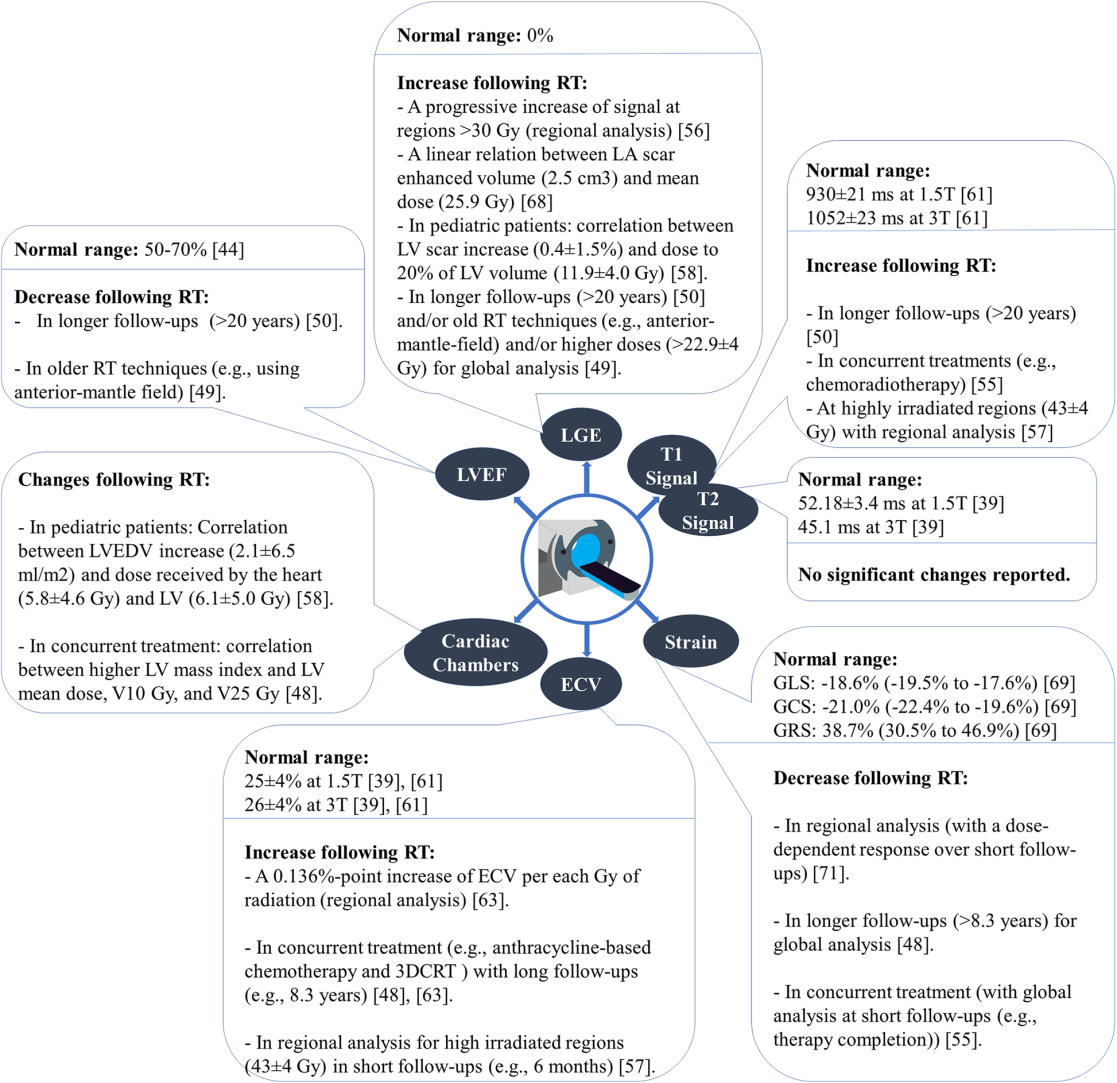
Summary of MRI-based measurements (LVEF, LGE, T1 signal, T2 signal, strain, ECV, and cardiac chamber dimensions), including normal ranges for healthy controls and changes following RT.

Importantly, unlike systemic therapy (e.g., chemotherapy), where a more homogeneous distribution of the drug is assumed over the entire heart, radiation dose distribution is clearly spatially heterogeneous, with local cardiac doses depending on the proximity of the tumor to the heart (and a sharp fall-off in dose outside of the treatment volume). Accurately accounting for these dose variations requires careful regional assessments of the heart for each unique patient and treatment plan. As a result, patient-specific multimodal image registration (from planning CT to MRI) will play a key role in accurately quantifying cumulative regional doses across the beating heart and determining local correlations between clinically useful MRI-based metrics and RT dose. In summary, a compact list of important findings is provided in Fig. 5.

## Conclusion and future recommendations

The findings in this review suggest that global MRI-based metrics primarily detect RT-induced MCT only after long follow-ups (e.g., >8 years), with the exception of pediatric patients that may show changes in relevant metrics over shorter follow-ups within 1-2 years. Since the radiation delivered during RT is patient-specific and highly spatially heterogeneous depending on the particular type and location of the tumor, the dose to regional cardiac structures is also heterogeneous. Thus, regional quantifications of both dose and relevant MRI-based metrics may be required for an accurate assessment of potential MCT. Furthermore, since the likelihood of recovery or mitigation of the effects of MCT decrease with time since undergoing RT, regional assessments of cardiac structure and function (e.g., T1 signal, ECV, LGE, strain) offer the potential of detecting early subclinical changes before changes in global metrics are measurable.

Some of the major regional myocardial findings on MRI currently reported in the literature include an increase of T1 signal at high-irradiated regions [57], decrease of regional strain with different dose-dependent responses over different segments [71], a linear relationship between segmental ECV increase and mean dose per segment (a 0.136% increase per Gy) [63], correlation between LV scaring and LV dose [58], and a progressive increase of LGE at regions receiving >30 Gy [56]. It should be noted that among all MRI metrics, myocardial T2 signal was the only parameter that did not show any changes or dose-dependency following RT.

To optimally detect these regional changes and quantify potential dose–effect relationships locally requires accurate image registration between planning CT (from which the clinical dosimetry map is derived) and various MR sequences that have the ability to detect local subclinical abnormalities. In conclusion, the continual advancement of quantitative cardiovascular MR techniques, the ability to plan and control precise spatiotemporal delivery of therapeutic radiation, and our growing understanding of the evolving effect of localized RT doses on regional and global cardiovascular dysfunction offers significant potential to improve the cardiovascular outcomes of the myriad patients undergoing radiotherapy each year by aiding in optimal therapeutic planning, the early diagnosis of impending cardiovascular toxicity, and the clinical evaluation/monitoring of novel interventions or alternate treatment regimens.

## Data Availability

Data sharing is not applicable to this article as no datasets were generated or analyzed during the current study.

## Abbreviations

3DRT: 3D Conformal Radiation Therapy
AAPM: American Association of Physicists in Medicine
AHA: American Heart Association
CMR: Cardiac Magnetic Resonance
CT: Computed Tomography
ECV: Extracellular Volume
GCS: Global Circumferential Strain
GLS: Global Longitudinal Strain
GRS: Global Radial Strain
HL: Hodgkin Lymphoma
IL: Interleukin
IMRT: Intensity Modulated Radiotherapy
LA: Left Atrium
LGE: Late Gadolinium Enhancement
LV: Left Ventricle
LVEF: Left Ventricle Ejection Fraction
MCT: Myocardial Toxicity
MOLLI: Modified Look-Locker Inversion Recovery
MRI: Magnetic Resonance Imaging
MUGA: Multiple-Gated Acquisition
NHL: Non-Hodgkin Lymphoma
PET: Positron Emission Tomography
PRISMA: Preferred Reporting Items for Systematic Reviews and Meta-Analyses
RA: Right Atrium
ROS: Reactive Oxygen Species
RT: Radiotherapy
RV: Right Ventricle
SSFP: Steady-State Free Precession
T1-VIBE: T1-Volumetric Interpolated Breath-hold Examination
TNF: Tumor Necrosis Factor
